# Interim Evaluation of Germany’s Sugar Reduction Strategy for Soft Drinks: Commitments versus Actual Trends in Sugar Content and Sugar Sales from Soft Drinks

**DOI:** 10.1159/000529592

**Published:** 2023-02-21

**Authors:** Peter von Philipsborn, Oliver Huizinga, Anna Leibinger, Diana Rubin, Jacob Burns, Karl Emmert-Fees, Sara Pedron, Michael Laxy, Eva Rehfuess

**Affiliations:** ^a^Chair of Public Health and Health Services Research, Ludwig-Maximilians-Universität München (LMU Munich), Munich, Germany; ^b^Pettenkofer School of Public Health, Munich, Germany; ^c^German Non-Communicable Disease Alliance (DANK), Berlin, Germany; ^d^Centre for Nutritional Medicine, Vivantes Humboldt-Klinikum, Berlin, Germany; ^e^Professorship of Public Health and Prevention, Technical University of Munich, Munich, Germany; ^f^Institute of Epidemiology, Helmholtz Zentrum München, Neuherberg, Germany

**Keywords:** Soft drinks, Sugar intake, Obesity, Germany, United Kingdom

## Abstract

**Introduction:**

A high intake of sugar, in particular from sugar-sweetened soft drinks, increases the risk for obesity, type 2 diabetes mellitus, and dental caries. Germany has pursued a national strategy for sugar reduction in soft drinks based on voluntary commitments by industry since 2015, but its effects are unclear.

**Methods:**

We use aggregated annual sales data from Euromonitor International to assess trends in mean sales-weighted sugar content of soft drinks and per capita sugar sales from soft drinks in Germany from 2015 to 2021. We compare these trends to the reduction path set by Germany’s national sugar reduction strategy and to data for the United Kingdom, which adopted a soft drinks tax in 2017 and which we selected as best practice comparison country based on pre-defined criteria.

**Results:**

Between 2015 and 2021, the mean sales-weighted sugar content of soft drinks sold in Germany decreased by 2% from 5.3 to 5.2 g/100 mL, falling short of an interim 9% reduction target and a 29% reduction observed in the United Kingdom over the same period. Sugar sales from soft drinks in Germany decreased from 22.4 to 21.6 g/capita/day (−4%) between 2015 and 2021 but remain high from a public health perspective.

**Conclusions:**

Reductions observed under Germany’s sugar reduction strategy fall short of stated targets and trends observed internationally under best practice conditions. Additional policy measures may be needed to support sugar reduction in soft drinks in Germany.

## Introduction

An increasing body of evidence links excess consumption of free sugars with a number of adverse health outcomes [1, 2]. Sugar intake from beverages is of particular concern [2]. Evidence from randomized controlled trials and observational studies shows that sugar-sweetened beverages can contribute to weight gain and an increased risk for overweight and obesity [3, 4, 5], while observational studies show positive associations with an increased risk for diabetes mellitus type 2, dental caries, and overall mortality [6, 7, 8]. Sugar-sweetened soft drinks are, therefore, considered an important driver of the global epidemic of obesity, type 2 diabetes mellitus, and other chronic diet-related diseases [9, 10].

The World Health Organization (WHO), therefore, recommends to limit intake of free sugars among adults and children to no more than 10% of total energy intake, noting that additional health benefits may be achieved by limiting it to no more than 5% [2]. Similarly, the European Food Safety Authority (EFSA) concludes that due to the observed health risks, no safe upper level of intake can be set for added and free sugars, and that intake should be as low as possible in the context of a nutritionally adequate diet [11]. The German guidelines on sugar intake follow the WHO in recommending to limit intake of free sugars to less than 10% of total energy intake, or approximately 50 g/day for an average adult with a total energy intake of 2,000 kcal/day [12]. Current sugar intake levels in Germany are estimated to range from 13% to 19% of total energy intake, depending on gender and age [12].

Sugar reduction in soft drinks is also a declared policy objective of the German government. As its landmark nutrition policy act, it announced in 2015 a National Strategy for the Reduction of Sugar, Fat, and Salt in Processed Foods [13]. In the subsequent years, specific reduction targets were defined through formal agreements between the government and food industry groups, including a commitment to reduce the average sugar content of soft drinks sold in Germany by 15% between 2015 and 2025 [14, 15, 16]. In 2022, the newly elected German government announced that if the prior approach based on voluntary commitments by the food industry proved insufficient, additional measures (including a tax on sugar-sweetened beverages) would be considered as part of a new national nutrition strategy to be developed until the end of 2023 [17, 18].

Against this backdrop, the present paper evaluates Germany’s current sugar reduction strategy for soft drinks by assessing trends in mean sales-weighted sugar content of soft drinks and per capita sugar sales from soft drinks from 2011 to 2021. We compare these trends with the reduction path set by Germany’s national sugar reduction strategy, and with data for the United Kingdom (UK), which adopted a soft drinks tax in line with international recommendations in 2017, and which we selected as best practice comparison country based on pre-defined criteria.

## Methods

### Study Design and Setting

This is a policy evaluation based on a repeat cross-sectional analysis of aggregated annual sales and ingredient data provided by Euromonitor International, a market research company. The evaluation is based on three comparisons: actual trends versus reduction targets; actual trends in Germany versus trends in the UK; and actual trends before and after Germany’s sugar reduction strategy was announced. We chose the UK as international best practice comparison country based on the following pre-defined criteria: geographical proximity and similarity in market size to Germany; and implementation of a soft drinks tax aligned with WHO recommendations (including the use of a tiered tax design to incentivize reformulation) [19]. A detailed description of our methodological approach, including the steps taken to select the comparison country, is provided in the online supplementary material (see www.karger.com/doi/10.1159/000529592 for all online suppl. material). Our study follows the STROBE reporting guideline [20].

### Variables

We assess the mean sales-weighted sugar content of soft drinks, the mean amount of sugar sold through soft drinks per capita per day, and mean soft drinks sales per capita and day. In line with common usage, we define soft drinks as non-alcoholic, non-dairy beverages with added sweeteners (including sugar and other caloric sweeteners, as well as high-intensity, non-nutritive sweeteners such as aspartame) [21]. Our definition of soft drinks, therefore, includes varieties with sugar as well as sugar- and calorie-free varieties sweetened with non-nutritive sweeteners. Sugar is defined in line with the EFSA definition of added sugars [11].

### Data Sources and Methods of Assessment

We use data from the Euromonitor Passport database collected and provided by Euromonitor International. Euromonitor provides sales and ingredient data based on primary and secondary data sources, including company reports, official statistics, store audits, product information (such as ingredient and nutrient declarations), interviews with companies, and estimates by in-house experts [22]. The Euromonitor Passport database is considered to be one of the most comprehensive and reliable sources for such data and has been used extensively in public health research, including studies on soft drinks sales and composition [23, 24, 25]. For soft drinks, the database covers both off-trade sales (i.e., sales through retail outlets) and on-trade sales (i.e., through hospitality and catering outlets). Euromonitor uses an internationally standardized methodology, which allows for comparisons between countries and over time [22].

We obtained sales and ingredient data for all beverage categories meeting our definition of soft drinks, i.e., carbonates (including cola carbonates, lemonade and lime, ginger ale, tonic water and other bitters, orange carbonates, and other non-cola carbonates), juice drinks (with up to 24% juice), nectars (with more than 24% but less than 100% fruit), flavoured bottled water, functional bottled water, energy drinks, sports drinks, and ready-to-drink tea. We included powder and liquid concentrates in our calculation of per capita sugar sales from soft drinks but not in the calculation of the mean sales-weighted sugar content and per capita soft drink sales. We aggregated data for the beverage and ingredient categories included in our definition of soft drinks and free sugars, respectively, as listed in the online supplementary material. For information on Germany’s sugar reduction strategy, we used official government publications [13, 15, 16, 26].

### Analysis

We descriptively plot the annual mean sales-weighted sugar content of soft drinks and per capita sugar sales from soft drink sales from 2011 to 2021. To compare this trend to the targets of Germany’s national sugar reduction strategy, we calculated a linear reduction path based on the observed value for the strategy’s baseline year (2015) and the relative reduction target set by the strategy for 2025 (the strategy does not define interim targets but emphasizes that its reduction targets will be achieved stepwise and gradually, justifying the assumption of a linear reduction path [14, 15]). We then compare outcome trends in Germany to those over the same period in the UK. Finally, we compare outcome trends in Germany before and after 2015. For this last comparison, we calculate the compound annual reduction rate in the mean sales-weighted sugar content of soft drinks in Germany for 2011–2015 and 2015–2021, respectively.

We use 2015 as the baseline for our analysis, as this is the baseline year to which the sugar reduction targets, as stated in government and industry publications, refer [14, 15]. 2015 is also the year in which the sugar reduction strategy was first publicly announced, even though the specific reduction targets for soft drinks were published only in 2019 (according to industry sources, the earlier baseline year of 2015 was chosen to account for sugar reductions achieved in the preceding years, i.e., between the first announcement of the strategy in 2015 and the publication on the 15% reduction target in 2019) [14]. We also report data for 2011–2014 to allow for a comparison of trends before and after the strategy’s baseline year. We chose 2011–2021 as the overall time frame of our analysis as this was the time span for which comparable data were available from Euromonitor when we conducted our analyses.

### Study Registration and Protocol Availability

A protocol for this study was developed and prospectively registered with the Open Science Framework (registration DOI 10.17605/OSF.IO/3WJ49) before data were analysed [27]. Differences between protocol and manuscript are explained in the online supplementary material.

## Results

### Trends in Sugar Content of Soft Drinks in Germany

**Fig. 1.**
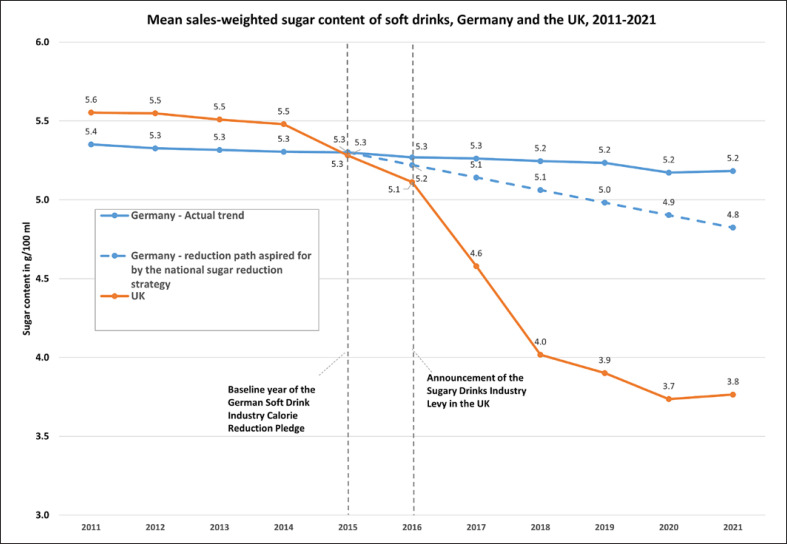
Mean sales-weighted sugar content of soft drinks in Germany and the UK, 2011–2021 in g/100 mL (solid lines), as well as the reduction path set by Germany’s national sugar reduction strategy (dashed line). Data sources: Own calculations based on data from Euromonitor International and Germany’s Federal Ministry of Food and Agriculture [16].

**Fig. 2.**
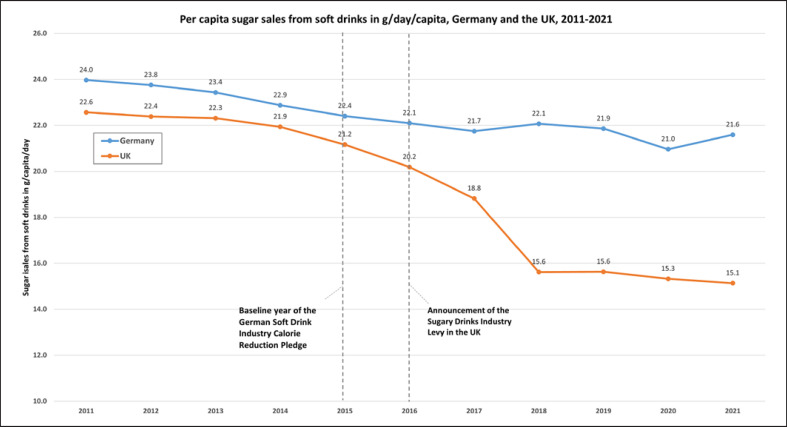
Mean sugar sales from soft drinks per capita in Germany and the UK, 2011–2021 in g/d/capita. Data sources: Own calculations based on data from Euromonitor International (Passport database).

**Fig. 3.**
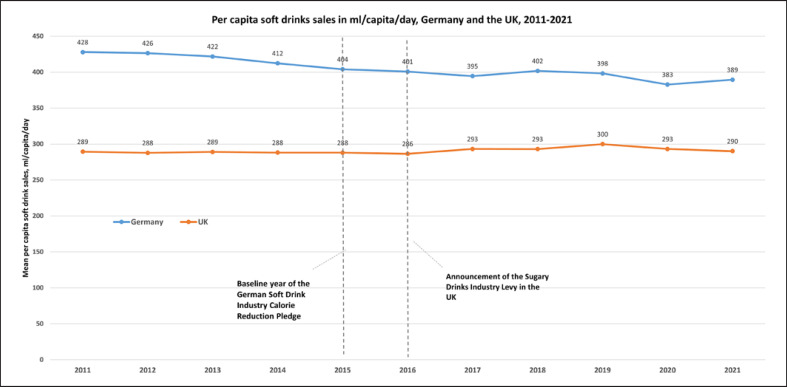
Mean soft drink sales per capita in Germany and the UK, 2011–2021 in mL/day/capita. Data sources: Own calculations based on data from Euromonitor International (Passport database).

The mean sales-weighted sugar content of soft drinks sold in Germany decreased between 2011 and 2021 (from 5.4 g/100 mL to 5.2 g/100 mL, −3%), as did mean per capita sugar sales from soft drinks (from 24 g/capita/day to 22 g/capita/day, −10%) and mean soft drinks sales per capita (from 428 mL/capita/day to 389 mL/capita/day, −9%) (see Table 1; Fig. 1, 2, 3).

### Comparison of Actual Trends in Germany with Reduction Targets and with Trends in the UK

During the time period covered by Germany’s national sugar reduction strategy for which data were available (2015–2021), the mean sales-weighted sugar content of soft drinks sold in Germany decreased by 2% (from 5.3 g/100 mL to 5.2 g/100 mL). This contrasts with a 9% interim reduction target for the same time period implied by the sugar reduction strategy, as well as with a 29% reduction (from 5.3 g/100 mL in 2015 to 3.8 g/100 mL in 2021) observed in the UK (see Fig. 1). Sugar sales from soft drinks decreased in the UK in this time period from 21 g/capita/day in 2015 to 15 g/capita/day in 2021 (−28%), while total soft drink sales increased slightly from 288 to 290 mL/capita/day (+1%) (see Table 1).

### Comparison of Pre- and Post-Pledge Trends

The compound annual reduction rate of the mean sales-weighted sugar content of soft drinks in Germany during the 4 years prior to the baseline of the sugar reduction strategy (2011–2015) was 0.2% and increased slightly to 0.4% during the years covered by the strategy for which data were available (2015–2021).

## Discussion

### Key Findings and Public Health Implications

During the time period covered by Germany’s current national sugar reduction strategy for which data were available (2015–2021), the mean sales-weighted sugar content of soft drinks sold in Germany decreased only slightly by 2%, which falls short of an interim 9% reduction target, as well as of the 29% reduction achieved in the UK during the same time period. At the current pace, Germany is, therefore, not on track for meeting the 15% reduction target it has set itself for 2025, which is modest compared to the reductions achieved in the UK to date. The average annual reduction rate increased slightly after the strategy was announced in 2015, from 0.2% per year in 2011–2015 to 0.4% per year in 2015–2021.

Per capita sugar sales from soft drinks in Germany decreased by 4% since the national sugar reduction strategy was first announced in 2015 but still stood at 22 g/day/capita in 2021. For an average adult with a daily energy requirement of 2,000 kcal/day, this corresponds to almost half the recommended maximum intake of free sugars (10% of total energy intake or 50 g/day) [2, 12]. Dietary surveys show that soft drink intake is highly unevenly distributed in the population, with children, teenagers, and young adults consuming two to three times more than older adults, and low socioeconomic status groups consuming more than high socioeconomic status groups [28, 29]. This suggests that young people and socioeconomically disadvantaged groups in Germany may exceed the recommended maximum intake of free sugars through their soft drink intake alone. This underlines the importance of reducing sugar intake from soft drinks.

Soft drinks sales per capita in Germany decreased during that same time period by 3.6% (from 404 mL/capita/day in 2015 to 389 mL/capita/day in 2021) but remain higher than recommended (due to their demonstrated adverse health effects, dietary guidelines generally do not define a safe upper limit for soft drinks, but recommend to avoid or limit their intake [30, 31]). Soft drink sales per capita slightly increased in the UK (from 288 mL/capita/day in 2015 to 290 mL/capita/day in 2021, +0.7%), suggesting that substantial sugar reductions do not necessarily result in lower total sales of soft drinks.

### Strengths and Limitations

To the best of our knowledge, our study is the most comprehensive assessment to date of recent trends in sugar content, sales, and sugar sales from soft drinks in Germany. The only publicly available recent assessments we are aware of were limited to comparisons between single years (2016 and 2018, and 2018 and 2019, respectively), did not cover soft drink sales in the hospitality sector, were based on non-representative samples, and were not sales-weighted [32, 33, 34]. The Euromonitor Passport Database used for our analysis provides a comprehensive market coverage and is based on a standardised methodology, which allows for comparisons between countries and across time [22]. Our analysis is based on sales and ingredient data, which are, unlike self-reported dietary survey data, not prone to recall and social desirability bias. Finally, we defined key aspects of our methodology in an a priori protocol developed and published before data were analysed [27].

Our study also has a number of limitations. While sales figures can be considered reasonable proxies for consumption and may be more reliable than self-reported dietary intake data, they do not account for food waste of the final consumer (i.e., drinks left over or discarded by consumers). Besides, we did not include liquid and powder concentrates (which are diluted by the final consumer before consumption) in our estimates for soft drink sales volumes and mean sugar content, as dilution ratios may vary. We calculated sugar content based on the use of sugar as ingredient, but were unable to account for the sugar content of fruit juices used as ingredient in some types of soft drinks (such as nectars). Due to data limitations, we were also unable to differentiate between regular and low-calorie soft drinks, and we did not assess trends in the use of high-intensity sweeteners. We were also unable to assess trends for sub-populations (such as children), as our data represents population-wide averages. Moreover, while Euromonitor is generally considered a reliable source of sales and ingredient data, its data are partially based on estimates by its technical and industry experts, and reported outcomes may, therefore, be different from the true values [22]. Due to data limitations we were unable to quantify this uncertainty. Finally, our analysis is descriptive, and we did not attempt to establish causal relationships between the observed trends and factors that may have influenced them. In particular, reductions seen in average sugar sales from soft drinks in Germany between 2015 and 2021 may reflect secular trends, rather than effects of the sugar reduction strategy. Of note, dietary survey data from the DONALD study suggest that among children and adolescents in Germany sugar intake from soft drinks decreased between 1985 and 2016 [35].

### Comparisons with Other Studies

Data on the sugar content of soft drinks, and sugar sales from soft drinks in Germany is limited. Following a mandate by Germany’s Federal Ministry of Food and Agriculture (BMEL), the Federal Research Institute for Nutrition and Food (Max-Rubner-Institut, or MRI) published two reports on the sugar content of soft drinks on the German market in 2018 and 2020 [34, 36]. The second and more comprehensive of these reports, published as an updated version in June 2020, reports data for two main beverage categories: soft drinks (“Erfri­schungsgetränke” in German) as well as sugar-sweetened beverages (“gesüßte Erfrischungsgetränke” in German, including soft drinks with caloric sweeteners but excluding soft drinks sweetened exclusively with non-nutritive sweeteners) [36]. Data for specific sub-categories (such as lemonades) are also reported. Data collection covered beverages sold through retail outlets, and followed a stepwise process including online research on manufacturers’ websites, enquiries with manufacturers as well as on-site research in grocery stores. Results are not weighted by sales, but for the follow-up assessment in 2019, data on the mean sugar content are presented separately for the full range of products included in the analysis, and for top-selling products identified through household panel data from the market research company GfK. For the full range of soft drinks, the median sugar content is reported as 6.2 g/100 mL in 2018, and 6.0 g/100 mL in 2019, a relative decrease of 3.2% [36]. For sugar-sweetened beverages, the median sugar content of the full product range is reported as 6.5 g/100 mL in 2018 and 6.2 g/100 mL in 2019, a relative decrease of 4.6% [36]. For top-selling products, the median sugar content for sugar-sweetened beverages is reported as 5.9 g/100 mL in 2019. In our analysis, we found the average sales-weighted sugar content of soft drinks to be 5.25 g/100 mL in 2018 and 5.23 g/100 mL in 2019, a relative decrease of 0.20%. Our figures, therefore, show a lower absolute level of sugar content for both years, and a smaller relative decrease between the 2 years. These differences may be explained by the fact that our figures are weighted by sales, include the hospitality sector, and are based on a slightly different definition of soft drinks (the MRI data set did not include nectars) and on a different data source (Euromonitor data vs. the MRI’s own sample of beverages). A comparison of our results with further studies (including studies from the UK) is provided in the online supplementary material.

### Policy Implications

So far, the approach pursued by the German government to reduce sugar intake from soft drinks and average sugar content of soft drinks sold in Germany has not fully achieved its stated objectives. This suggests that additional policy measures may be needed. In 2020, the Scientific Advisory Council at Germany’s Federal Ministry of Food and Agriculture (WBAE) proposed a number of measures to reduce the adverse health effects of soft drink consumption in Germany, including a levy on sugar-sweetened beverages proportional to their content of free sugars [37]. Besides its intended effects on sales and consumption of sugar, this could generate revenue of 1.0–1.9 billion € annually, which could be used to partially fund a value added tax exemption for healthy foods including fruit and vegetables [37]. This proposal has received renewed attention in light of recent increases in the price of staple foods, as well as due to its potential environmental co-benefits [38]. Similar to the Sugary Drinks Industry Levy in the UK, revenue could also be used to fund free, healthy school meals [37]. Further measures recommended by the WBAE include improvements to the availability of healthy beverages in schools, kindergartens, hospitals, and other public settings and an action plan for the promotion of drinking water (including a mandate that free drinking water must be available for consumption in all foodservice establishments) [37]. These recommendations are in line with a report of Germany’s national nutrition research institute (the Max-Rubner-Institute), which concluded in 2016 that regulatory and fiscal measures should be considered if the industry’s voluntary reformulation commitments proved insufficiently effective [39]. Additional measures recommended by the institute include improved nutrition labelling and the regulation of marketing of food with a high content of sugar [39]. In light of the findings of the present study, and the well-established adverse health effects of sugar-sweetened soft drinks, these measures should be considered as part of the new national nutrition strategy announced for 2023 [18].

## Statement of Ethics

No human subjects were involved in this research, and no ethical clearance was required according to the regulations of the Ethics Committee of Ludwig-Maximilians-Universität München (LMU Munich).

## Conflict of Interest Statement

PvP has received research funding from Germany’s Federal Ministries of Food and Agriculture (BMEL), Education and Research (BMBF) and Environment and Consumer Protection (BMUV), as well as travel cost reimbursements and speaker and manuscript fees from the German and Austrian Nutrition Societies (DGE and ÖGE), among others. ER has received research funding from BMEL and BMBF. ML has received research funding from BMBF. OH is an employee of the German Diabetes Society (DDG) and the German Obesity Society (DAG) and has previously been an employee of foodwatch. The other authors have no conflicts of interest to declare.

## Funding Sources

The project was supported by funds from members of the German Non-Communicable Disease Alliance, including the following: Federal College of Paediatricians, German Obesity Association, German Paediatric Association, German Diabetes Association, German Heart Foundation, Association of Diabetes Advisors and Trainers, German Association for Nutritional Medicine and German Association for Social Medicine and Prevention. Support came also from staff positions at Ludwig-Maximilians-Universität München (LMU Munich) and Technical University Munich (TUM).

## Author Contributions

P.v.P., O.H., A.L., D.R., J.B., K.E.F., S.P., M.L., and E.R.: methodology, writing – review and editing, and conceptualization; P.v.P. and A.L.: data curation and formal analysis; A.L.: validation; P.v.P.: writing – original draft; O.H. and P.v.P: funding acquisition.

## Data Availability Statement

The Euromonitor International Passport data used in this research is proprietary data owned by Euromonitor International, a market research company. Access to the database has to be acquired from Euromonitor International and is generally subject to a fee. Further enquiries can be directed to the corresponding author.

## Supplementary Material

Supplementary data

## Figures and Tables

**Table 1:** Mean sales-weighted sugar content of soft drinks, soft drink sales, and sugar sales from soft drinks, Germany and the UK, 2011-2021

Country	Measure	Unit	2011	2012	2013	2014	2015*	2016**	2017	2018***	2019	2020	2021	Change 2011–2021	Change 2015–2021
Germany	Mean sales-weighted sugar content of soft drinks (excluding concentrates)	g/100 mL	5.4	5.3	5.3	5.3	5.3	5.3	5.3	5.2	5.2	5.2	5.2	**–3.2%**	**–2.2%**

Germany	Total sugar sales through soft drinks (including concentrates)	g/capita/day	24.0	23.8	23.4	22.9	22.4	22.1	21.7	22.1	21.9	21.0	21.6	**–9.9%**	**–3.6%**

Germany	Total soft drink sales (excluding concentrates)	mL/capita/day	428	426	422	412	404	401	395	402	398	383	389	**–9.0%**	**–3.6%**

UK	Mean sales-weighted sugar content of soft drinks (excluding concentrates)	g/100 mL	5.6	5.5	5.5	5.5	5.3	5.1	4.6	4.0	3.9	3.7	3.8	**–32.2%**	**–28.7%**

UK	Total sugar sales through soft drinks (including concentrates)	g/capita/day	22.6	22.4	22.3	21.9	21.2	20.2	18.8	15.6	15.6	15.3	15.1	**–32.9%**	**–28.5%**

UK	Total soft drink sales (excluding concentrates)	mL/capita/day	289	288	289	288	288	286	293	293	300	293	290	**0.2%**	**0.7%**

Data sources: Own calculations based on data from Euromonitor International (Passport database). UK, United Kingdom; SDIL, Sugary Drinks Industry Levy.

* Baseline year of the reduction targets of Germany's national sugar reduction strategy for soft drinks.

** Year of the announcement of the Sugary Drinks Industry Levy (SDIL) in the UK.

*** Year when the SDIL took effect in the UK.
