# Supplementary material for: Interim Evaluation of Germany’s Sugar Reduction Strategy for Soft Drinks: Commitments versus Actual Trends in Sugar Content and Sugar Sales from Soft Drinks
Source: Ann Nutr Metab. 2023 Feb 21;79(3):282–90. doi: 10.1159/000529592 (PMC10568594; doi:10.1159/000529592)
Supplement: Supplementary file 1 — Supplementary data [file anm-0079-0282-s01.docx]

**Supplementary material**

**Supplementary material to:** von Philipsborn P, Huizinga O, Leibinger A, Rubin D, Burns J, Emmert-Fees K, Pedron S, Laxy M, Rehfuess E: *Evaluation of Germany’s Sugar Reduction Strategy for Soft Drinks: Commitments versus Actual Trends in Sugar Content and Sugar Sales from Soft Drinks.* Manuscript submitted to Annals of Nutrition and Metabolism, 2022.

**Table of content**

[**1.** **Methods** 2](#_Toc117141411)

[**1.1.** **Selection of the comparison country** 2](#_Toc117141412)

[**1.2.** **Data sources, methods of assessment and calculation of variables** 4](#_Toc117141413)

[**2.** **Category definitions** 5](#_Toc117141414)

[**2.1.** **Beverages** 5](#_Toc117141415)

[**2.2.** **Ingredients** 10](#_Toc117141416)

[**3.** **Comparison with other studies** 12](#_Toc117141417)

[**3.1.** **Studies from Germany** 12](#_Toc117141418)

[**3.2.** **Studies from the UK** 13](#_Toc117141419)

[**4.** **Generalizability** 13](#_Toc117141420)

[**5.** **Data sources used by Euromonitor** 14](#_Toc117141421)

[**5.1.** **Germany** 14](#_Toc117141422)

[**5.2.** **UK** 20](#_Toc117141423)

[**6.** **Differences between protocol and manuscript** 26](#_Toc117141424)

[**7.** **STROBE-nut Checklist** 28](#_Toc117141425)

[**References** 35](#_Toc117141426)

1. **Methods**
   1. **Selection of the comparison country**

We selected our comparison country (the United Kingdom, or UK) based on the following pre-defined criteria: i) implementation of a soft drinks tax during the past 10 years; ii) alignment of the tax design to WHO recommendations for soft drink taxes (see eText box 1) [1]; iii) geographic proximity to Germany (including location within Europe); and iv) similarity in market size to Germany. To identify potential comparison countries, we searched online inventories of countries with soft drinks taxes [2-6], and compiled a list of European countries with soft drinks taxes showing key tax design characteristics (see eTable 1), and compared these to the pre-defined selection criteria and decided through discussion in the research team on the selection of the comparison country.

We identified 10 European countries with soft drinks taxes in effect as of July 2022. Based on our pre-defined criteria we restricted our selection of comparison country to countries with a tiered tax. Of these, we excluded Poland, as it has implemented its tax in 2021 only, i.e. the last year for which data was available. We selected France and the UK over Ireland and Portugal due to their more comparable size and geographic proximity to Germany. We finally chose the UK over France due to the closer alignment of its tax design with current recommendations. In particular, its tax rate is closer to the recommended rate of at least 20% of the average price of the taxed beverage, and the revenue generated has been earmarked for health promotion measures when the levy was first announced [7].

| **eText box 1: WHO recommendations for the design of soft drinks taxes**   - Use of a specific excise tax (i.e. a tax that charges a specific amount per quantity of the product or ingredient), in contrast to a sales or value-added tax (i.e. a tax calculated as percentage of the product’s price), to avoid substitution effects to cheaper products with similarly harmful health effects. - Calculation of the tax rate based on the sugar content of soft drinks (e.g. use of a tiered tax with a tax rate that increases with the sugar content, in contrast to a flat taxes where the same tax rate is applied to all soft drinks), to incentivize reformulation by industry and the selection of soft drink varieties with lower sugar content by consumers. (This recommendation applies primarily to countries with a strong tax administration.) - Use of a tax rate that is sufficiently high to raise the retail price of soft drinks by 20% or more, to realize meaningful net reductions in caloric intake and consequent health effects. - Dedication of some or all of the revenue generated by the tax to health-related measures (e.g. the health care system or health promotion), to realize additional health gains and to increase public support.   Source: *Fiscal policies for diet and the prevention of noncommunicable diseases,* WHO 2016 [1]. |
| --- |

| **eTable 1: European countries with softs drink taxes as of July 2022** | | | | |
| --- | --- | --- | --- | --- |
| **Country** | **Tax type** | **Tax design** | **Date of introduction** | **Date of revision** |
| **Belgium** | Flat tax | €0.068 per L ($0.08) excise on soft drinks with added sweeteners; €0.41/L ($0.46) and €0.68/100 kg ($0.77) excise on liquid and powder concentrates, respectively | January 2016 | n.a. |
| **Finland** | Flat tax | € 0.22 per l ($0.25) on sugar-containing soft drinks and €0.12/L ($0.14) on sugar-free soft drinks and mineral waters | 1940 | 2011 |
| **France** | Tiered tax | Since 2018: Sliding tax scale with €3.63 for 2 kg of added sugar per hl of drink. Exemplary tax rates: €0.055/l on drinks with 5g total sugar/100 ml, €0.135/l on drinks with 10 g total sugar/100 ml. Beverages with non-calorically sweetened drinks are taxed at 3 €/hl. Drinks containing both sugars and sweeteners are subject to both taxes simultaneously.  From January 2012 to 2018: flat tax of €0.11 per 1.5 L ($0.08 per L) on drinks with added sugars or artificial sweeteners. | January 2012 | July 2018 |
| **Hungary** | Flat tax | HUF 7 per L ($0.02) on soft drinks; HUF 200 /L ($0.62) on syrup concentrates. | 2011 | n.a. |
| **Ireland** | Tiered tax | €0.20 per L ($0.23) on drinks with >5 g total sugar/100 mL; €0.30 per L ($0.34) on drinks with >8 g total sugar/100 mL. | May 2018 | n.a. |
| **Latvia** | Flat tax | €0.074 per L ($0.08) excise on drinks with added sugar, sweetener, or other flavoring (excludes fruit/vegetable juices with <10% added sugar and flavored/functional waters without added sugars, sweeteners, or flavorings).  Implemented May 2004; increased tax rate 2016. | May 2004 | 2016 |
| **Poland** | Tiered tax | PLN 0.5 per L ($0.12) on soft drinks with added sweeteners, caffeine, or taurine; PLN 0.05 ($0.01) extra fee per gram of sugar >5 g/100 mL; PLN 0.09 per L ($0.02) for drinks containing caffeine or taurine. Total soda fee cannot exceed PLN 1.2 ($0.32). Excludes sports or juice drinks with <5g sugar/100mL and dairy drinks. Drinks with >20% juice content and >5g sugar/100mL are not charged the base fee of PLN 0.5 per L. | January 1, 2021 | n.a. |
| **Portugal** | Tiered tax | €0.01 per L ($0.01) on drinks with <25 g sugar per liter; €0.06 per L ($0.07) on drinks with 25 to <50 g sugar per L; €0.08 per L ($0.09) on drinks with 50 to <80 g sugar per L; or €0.20 per L ($0.23) on drinks with ≥80 grams sugar per L. Applies to non-alcoholic beverages; exempts milks (cow or substitute) and 100% juices | February 2017 | 2018 |
| **Spain** | Flat tax | National tax: 21% VAT (increase from 10%) on drinks containing added natural and derived sweeteners and/or sweetening additives, excluding dairy. Regional tax in Catalonia: €0.12 per L ($0.14) levy for drinks with added sugars and >8 g sugar/100 mL, or €0.08 per L ($0.09) for 5–8 g sugar/100 mL, implemented in May 2017. | January 2021 | n.a. |
| **United Kingdom** | Tiered tax | £0.18 per L ($0.24) on drinks with 5–8 g total sugar/100 mL; £0.24 per L ($0.28) on drinks with >8 g total sugar/100 mL. Exempts dairy drinks (>75% dairy) and 100% fruit/vegetable juices. | April 2018 | n.a. |
| **Countries in the EEA + UK and Switzerland without SSB taxes:** Austria, Bulgaria, Croatia, Cyprus, Czech Republic, Denmark, Estonia, Germany, Greece, Iceland, Italy, Liechtenstein, Lithuania, Luxembourg, Malta, Netherlands, Norway (previously existing tax abolished with effect on 1 January 2021), Romania, Slovakia, Slovenia, Sweden and Switzerland. **Data sources**: [2-6] | | | | |

- 1. **Data sources, methods of assessment and calculation of variables**

The following paragraphs provide additional information on our data sources, methods of assessment and calculation of variables, which complement the information provided in the methods section of the main manuscript.

***Data sources and methods of assessment***

In our analysis, we used data from the ‘Industries’ section of the Euromonitor Passport database [8]. To assess sales volumes, we used figures for total volume market size. This includes both off-trade sales (i.e. sales through retail outlets, such as supermarkets, non-grocery retailers, vending machines, internet retailing and home delivery services) and on-trade sales (i.e. through hospitality and catering outlets, such as restaurants, bars, cafés and hotels). It does not include office and institutional sales, e.g. beverages available from dispensers in offices. To assess sugar sales from soft drinks we used data from the Euromonitor ‘Ingredients’ database. This database contains data on ingredient volumes based on end-product sales to consumers through the sales channels mentioned above. Ingredient volume data is calculated by Euromonitor based on sales data, ingredient declarations on the packaging, and, where no complete declarations are available, on patent literature, trade interviews and the knowledge of its technical specialists. The data measures the amount of all ingredients in products purchased at retail or foodservice channels by consumers, and does not include industrial or institutional applications [8]. To assess the mean sales-weighted sugar content of soft drinks, we matched data from the market size and the ingredient volume database. A list of sources used by Euromonitor to derive its soft drinks data is provided in section 5 of this supplementary annex.

We included all beverage categories meeting our definition of soft drinks, as described in the main manuscript. Of note, the Euromonitor Passport database contains data for an aggregate category labelled “Soft drinks”. This category is based on a wider definition of the term than ours, and also includes bottled water (we include only flavoured and functional bottled water) and ready to drink coffee. We therefore extracted data for all lower-level beverage categories meeting our definition of soft drinks, and aggregated these to construct an aggregate category of soft drinks limited to these.

Regarding ingredients, we included all ingredients in the “Sugars and bulk sweeteners” category of the Euromonitor Ingredient database, except for inositol, which is a sugar alcohol not included in the WHO definition of free sugars [9]. Specifically, we included sucrose, fructose, dextrose, glucose/fructose syrup, glucose/corn syrup, high fructose corn syrup and invert sugar. In the Euromonitor Passport database, ingredients are reported in metric tonnes. For glucose/corn syrup, glucose/fructose syrup and for high fructose corn syrup we conservatively assumed an average sugar content of 70% (the German Directive on Sugars for Human Consumption stipulates that glucose syrup must have a dry mass of at least 70% [10]; the dry mass of glucose-fructose syrup is not regulated by the Directive, but glucose-fructose syrup available on the German market typically has a dry mass of between 75-85% [11]). This is a conservative assumption which is likely to underestimate the sugar content and sugar sales from soft drinks, as volumes of glucose/corn syrup, high fructose corn syrup and glucose/fructose syrup in ingredient declarations may refer to the sugar content of syrups only, excluding the water content.

Section 2 of this supplementary appendix provides an overview on, and definitions of all beverage and ingredient categories included in our analysis.

For population figures, we used the World Bank population database [12].

***Calculation of variables***

We calculated total annual sales of soft drinks by aggregating the total annual sales volumes of the six beverage categories included in our definition of soft drinks. We calculated total sugar volume sold through soft drinks by aggregating the total volume of sugars included in our definition used as ingredients for the soft drinks included in our definition. For glucose/fructose syrup, glucose/corn syrup and high fructose corn syrup we applied a multiplier of 0.7 to account for the water content of these, as discussed above. To calculate the mean sales-weighted sugar content of soft drinks, we divided total annual sugar volume sold through soft drinks by the total annual sales volume of soft drinks. To derive per capita figures, we divided total sales and volume figures by the population size of the respective country in the respective year. Analyses were done in Microsoft Excel 365, version 2022.

1. **Category definitions**
   1. **Beverages**

Category definitions were provided by Euromonitor International

| **eTable 2: Included and excluded beverage categories and their definitions** | | | |
| --- | --- | --- | --- |
| **Hierarchy**  **level** | **Passport Category** | **Included?** | **Category definition** |
| 0 | Softdrinks | No | This is the aggregation of the following categories; Carbonates, Fruit/vegetable juice, Bottled water, Functional drinks, Concentrates, RTD tea, RTD coffee and Asian speciality drinks. |
| 1 | Bottled Water | No | This category is the aggregation of still bottled water (spring, mineral & purified), carbonated bottled water (spring, mineral & purified), flavoured bottled water and functional bottled water. Bulk bottled water is split out separately. It is defined as packaged drinking water – purified, spring or mineral – that is packaged in a container of 8 litres or larger. The bulk bottled water data types apply to Still Bottled Water and Carbonated Bottled Water but do not apply to functional or flavoured bottled water. Bulk water is classified according to the new Bulk retail volume and Bulk retail value data type regardless of channel of sale: bulk water sold via retail locations and bulk water delivered direct to a consumer’s home is included. Bulk water sold to institutions (offices, schools etc.) remains excluded. |
| 2 | Flavoured Bottled Water | Yes | Combined total of, both sparkling and still flavoured bottled water. However, functional flavoured or functional carbonated water is tracked in functional water. Commonly fruit juice or essence has a content of one milligram per litre or less. Fruit essence is sometimes referred to as ‘natural flavours’. Products with significant percentage juice content (often listed on the packaging) should be included in Juice Drinks (up to 24% juice). In addition, flavoured bottled water does not normally contain colourings or significant amounts of added sweeteners. So-called ‘infusions’ – still water containing small pieces of fruit for added flavour – would be tracked here. |
| 2 | Functional Bottled Water | Yes | This subsector utilises production techniques which go beyond typical water purification processes. Functional bottled water will often include added vitamins, minerals, fruits or herbs. This category includes all functional bottled water, both carbonated and still. The subsector will primarily include nutraceutical or fortified waters, where various types of fruit or herbal concentrate or vitamin and mineral extracts are added to the bottled water for nutrient value. The product typically carries added calcium and vitamins, or added herbs, such as ginseng or gingko biloba. ”Fitness” waters, which typically contain electrolytes and amino acids as well as added vitamins and minerals, are included here. Sport and Energy drinks are excluded. These products often do not contain colouring, and tend to be a lighter drink than sports drinks such as Gatorade, which often feature higher added sugar or electrolyte content. Leading brands in off-trade volume include, SmartWater, and Propel Fitness Water. Products with both significant amounts of carbohydrate (such as dextrose or glucose) and electrolyte, and which are marketed as recovery beverages should be tracked in Sports Drinks, regardless of product name. |
| 1 | Carbonates | Yes | Sweetened, non-alcoholic drinks containing carbon dioxide are included here. All carbonated products containing fruit juice (“sparkling juices”) are included here, unless they are tea-based (these are included in carbonated RTD tea) or carbonated Energy drinks, which are included in Energy Drinks. Carbonated bottled water is also excluded. Carbonates are an aggregation of cola carbonates and non-cola carbonates, whether regular or low calorie. Euromonitor International includes both naturally and artificially-sweetened carbonates. |
| 2 | Cola Carbonates | Yes* | This is the aggregation of regular cola and low-calorie cola carbonates. Colas generally include some combination of caramel colour, caffeine, and a sweetener. While vanilla and the kola nut were originally used to provide the characteristic flavour and caffeine content, other flavouring and caffeinating ingredients are now commonly used. |
| 3 | Low Calorie Cola Carbonates | Yes* | All products that have, and which are marketed on the basis of having, lower calorie content than regular cola carbonates are included here. This is regardless of whether they contain artificial sweeteners, sugar, both or alternatively neither. This is the aggregation of standard low calorie cola carbonates and speciality low calorie cola carbonates. |
| 3 | Regular Cola Carbonates | Yes* | This is the aggregation of standard regular cola carbonates and speciality regular cola carbonates. |
| 2 | Non-Cola Carbonates | Yes* | Refers to carbonated soft drinks which are not cola carbonates, including carbonated fruit juice. However, all carbonated waters, RTD teas, sports drinks, energy drinks, and RTD coffees are excluded. This is the aggregation of lemonade/lime, orange, mixers and other non-cola carbonates. Brewed soft drinks, such as Fassbrause or maltas, are included here. |
| 3 | Lemonade/Lime | Yes* | This is the aggregation of juice-based and non juice-based lemonade-lime non-cola carbonates. |
| 3 | Ginger Ale | Yes* | Carbonated beverage made with ginger or ginger flavouring. Common varieties are Canada Dry, Schweppes and Seagram’s. |
| 3 | Tonic Water/Other Bitters | Yes* | Tonic water is a carbonated beverage that derives its distinct bitter taste from the addition of quinine. This also includes tonic waters that are lightly flavoured (for example tonic water with lemon). Common varieties are Canada Dry, Schweppes and Seagram’s. Tonic water with lemon or lime or orange flavour added is known as bitter lemon or bitter lime or bitter orange, respectively. Also includes non-alcoholic carbonated bitter appertifs such as Crodino or Sanbitter. Common brands are Canada Dry and Schweppes. |
| 3 | Orange Carbonates | Yes* | This is the aggregation of juice-based and non juice-based orange non-cola carbonates. |
| 3 | Other Non-Cola Carbonates | Yes* | Includes all carbonated soft drinks that are not included in regular cola carbonates, low-calorie cola carbonates, lemonade-lime, orange or mixers. Products featuring flavour mixes, such as orange-pineapple, are included here. Leading brands in off-trade volume include Mountain Dew, Dr Pepper and Shasta. |
| 1 | Concentrates | Yes** | This is the aggregation of liquid concentrates and powder concentrates. |
| 2 | Liquid Concentrates | Yes* | Concentrates and syrups, or alternatively known squashes or dilutables, which are diluted with water before consumption. Based commonly on fruit juices, however are also available as other in other forms, for example cola. Dilution ratios vary from country to country, due to local preferences and available brands. Leading brands in off-trade volume include Robinsons, Tucan and Brookes. |
| 2 | Powder Concentrates | Yes* | Powder concentrates, including granules and blocks/bars/cubes are diluted with water before consumption. Please note that powdered ice teas are included within this subsector. Leading brands in off-trade volume include Tang, Nestea Instant Iced Tea Mix and Nestlé Orange-C. |
| 1 | Juice | No | This category covers all still packaged juice obtained from fruits or vegetables by mechanical processes, reconstituted or fresh, often including pulp or fruit/vegetable puree. All unpackaged juices are excluded. Only still drinks are included here. Carbonated varieties are included non-cola carbonates. Juice-flavoured milk drinks and fruit shakes which are primarily milk are excluded–these are instead tracked in Packaged Foods Dairy. However, if the juice component is greater, the product is to be excluded from Packaged Foods Dairy coverage and tracked under the relevant category (based on % juice content) within Soft Drinks juice. This sector is the aggregation of 100% juice, nectars (25-99% juice content), juice drinks (up to 24% juice content), and coconut & other plant waters. |
| 2 | 100% Juice | No | This is the aggregation of not from concentrate 100% juice, reconstituted 100%juice and frozen 100% juice. |
| 3 | Not from Concentrate 100% Juice | No | 100% pure fruit or vegetable juice that has not been reconstituted from concentrate. These products are commonly freshly-squeezed and stored within chilled cabinets. Not from concentrate juices can be partially pasteurised, which means their shelf life can range from four days to three weeks. Leading brands in off-trade volume include Tropicana Pure Premium Orange Juice, Florida’s Natural Orange Juice and Simply Orange. |
| 3 | Reconstituted 100% Juice | No | 100% pure fruit or vegetable juice (still) that has been reconstituted from concentrate. These products are normally ambient/room temperature. They usually have an extended shelf life, commonly up to 12 months. Leading brands in the off-trade include Minute Maid Premium Orange Juice, Dole and Fruktovy. |
| 2 | Juice Drinks (up to 24% Juice) | Yes | Includes all still juice drinks made up of fresh juice or concentrate, not exceeding 24% juice content. Leading brands in off-trade volume include Minute Maid, Capri Sun and President. |
| 2 | Nectars | Yes | This category includes all frozen and unfrozen juices that are manufactured using a base of concentrated juice or a pasteurised purée of the fruit pulp, to which sugar and water are/can be added. For citrus fruits, the fruit content of nectars is usually over 50%, however can go as low as 25% for other fruits. Leading brands in off-trade volume include Hui Yuan, Ocean Spray and Nongfu Orchard. |
| 2 | Coconut and Other Plant Waters | No | This category contains packaged beverages fully or partially derived from the liquid contained in coconuts or root-based plants/vegetables. In addition to coconut water, other examples include birch water, cactus water, artichoke water, maple water and other light, clear liquid beverages derived from water-bearing plants. In general, these products are lighter and can be consumed for hydration or sometimes post-athletic recovery (in contrast to thicker, heavier juice products). Coconut milk and plant-based dairy substitutes are excluded. Some coconut or other root plants are combined with other fruits in blended juice beverages. If coconut water or other plant waters represent the largest ingredient in the juice combination (70% coconut water v 30% orange juice, for example), the product will be tracked here. Otherwise, the product will be tracked in Nectars or Juice Drinks. Unpackaged coconut water and other unpackaged plant waters are excluded. Brand examples: Vita Coco, Innocent coconut water. |
| 1 | Energy Drinks | Yes | These are functional drinks designed to boost energy levels. Often lightly carbonated, these contain high levels of caffeine and a number of added water-soluble vitamins, most often a selection of B vitamins including niacin, pantothenic acid, vitamin B6, and vitamin B12. Ingredients can also include amino acids such as taurine and glucuronolactone, as well as herbal products such as guarana and ginseng. Can be carbonated or still. Leading brands in off-trade volume include Red Bull, Monster and RockStar. Glucose-based energy beverages such as Lucozade Energy are included here. |
| 2 | Reduced Sugar Energy Drinks | Yes* | Includes products with lower sugar content compared to ‘standard’ offering, and is positioned on the basis of being ‘better for you’/reduced sugar. A sugar-reduced product will typically contain artificial sweeteners in addition or instead of sugar, and as a result the total sugar/calorie content is lower compared to what is considered ‘standard’ |
| 2 | Regular Energy Drinks | Yes* | Energy drinks not meeting the definition of “reduced sugar energy drinks” provided above. |
| 1 | Sports Drinks | Yes | The choice of sports drink usually depends on the provision of fluids, carbohydrates or both. Included into this subsector are isotonic, hypotonic and hypertonic sports drinks. Isotonic are products that replace lost body fluids, electrolytes (sodium, potassium and chlorides) and glucose in similar concentrations to existing body fluid without causing either swelling or shrinkage of cells. These products usually contain about 5-8% carbohydrate and are intended to be consumed during exercise and/or heat exposure. Hypotonic this product is a weaker solution than your body fluid. These drinks contain less carbohydrate and therefore have lower osmolality (fewer dissolved particles than blood). These drinks help the body to speed up water absorption and are best used when you need urgent fluid replacement, as in after exercise. These drinks are not the best for energy replacement. Hypertonic - this drink is a stronger solution than your body fluid. These drinks are designed to replace and maintain energy levels during exercise of at least one hour. They are absorbed slowly and therefore are not appropriate for fluid replacement. Leading brands in off-trade volume include Gatorade, Powerade and Aquarius. |
| 1 | RTD Coffee | No | Includes packaged ready-to-drink coffee, consumed either hot or cold, made using a base of either brewed coffee or coffee extract. Excludes all coffee flavoured milk drinks that primarily target children, or where coffee is one of a number of flavours within the brand range. Leading brands in off-trade volume include Georgia, Nescafé and Suntory Boss. |
| 1 | RTD Tea | Yes | This category includes all packaged products based on brewed tea or tea extract. May be sweetened or unsweetened, carbonated or still, with a wide variety of different flavourings. May contain juice. |
| 2 | Carbonated RTD Tea and Kombucha | Yes* | Carbonated packaged ready-to-drink tea, this does not include leaf or powdered tea. Naturally carbonated RTD Teas, such as kombucha, are included here. Leading brands in off-trade volume include Lipton Ice Tea Sparking, Rosynka and TEBS. |
| 2 | Still RTD Tea | Yes* | Non-carbonated packaged ready-to-drink tea this does not include leaf or powdered tea. Leading brands in off-trade volume include Master Kong Green Tea, Lipton and Nestea Lemon Tea. |
| 2 | Asian Speciality Drinks | No | This category includes all traditional Asian drinks not included in RTD tea or juice drinks, including products such as Bandung (rose syrup with milk), bird’s nest, tamarind juice, ginger, lemongrass, roselle, zalaka, jelly drinks including grass jelly (cincau), sugar cane, and vinegar drinks. Lactic acid drinks, such as Calpis, are included here. Drinks containing a limited amount of yogurt (generally 3% or less) such as Bikkle, are included here, though drinking yogurts such as Yakult are excluded. While both products are highly popular in markets like Japan, drinking yogurts will contain mostly yogurt with a very short shelf life (two weeks or less), while yogurt drinks will contain less than 3% dairy and remain on the shelves for up to 9 months. All nut or pulse-based products, such as peanut milk, almond juice, or soy drinks are tracked in Non-Dairy Milk alternatives in Passport Packaged Food. |
| *Included as part of the category of next higher hierarchy level (e.g. “Cola carbonates” are included in our analysis as part of the category “Carbonates”); **Included in our calculation of total sugar sales through soft drinks, but excluded from mean sales-weighted sugar content of soft drinks and total soft drink sales. Data sources: Euromonitor International (Passport database). | | | |

- 1. **Ingredients**

Category definitions were provided by Euromonitor International.

| **eTable 3: Included and excluded sugar-containing ingredient categories and their definitions** | | | |
| --- | --- | --- | --- |
| **Hierarchy level** | **Passport category** | **Included?** | **Category definition** |
| 2 | Sugars and Bulk Sweeteners | No | These are the nutritive or bulk sweeteners, which add bulk and texture to food and drink as well as sweetening capability. The category includes simple sugars (monosaccharides), processed sugars and syrups and sugar alcohols (derived from the hydrogenation of sugar and syrups). |
| 3 | --Dextrose | Yes | Dextrose, an isomer of glucose, is a simple and naturally occurring sugar and one of the main two sugars found in honey. It is produced commercially by the enzymatic hydrolysis of starch. Main product categories include soft drinks, baked goods and snack bars. |
| 3 | --Fructose | Yes | A simple sugar (monosaccharide) found in honey, fruits and vegetables. |
| 3 | --Glucose/Corn Syrup | Yes* | A naturally occurring, simple sugar (monosaccharide), glucose is produced commercially through enzymatic hydrolysis of starch. As corn syrup, it is most widely used as a thickener or humectant and is also used in conjunction with high intensity sweeteners. Key product categories include confectionary, dairy products and ice cream. |
| 3 | --Glucose/Fructose Syrup | Yes* | A naturally occurring, simple sugar (monosaccharide), glucose is produced commercially through enzymatic hydrolysis of starch. As fructose syrup, it is most widely used as a replacement for sugar in dairy products, ice cream and biscuits. |
| 3 | --High Fructose Corn Syrup | Yes* | This is derived from corn syrup that has been enzymatically treated by the enzyme glucose isomerase to increase the fructose content before being mixed with pure corn syrup (100% glucose). This process increases sweetness and HFCS has the same sweetness as sugar. It is mainly used in soft drinks, but also dairy products and jams and preserves. |
| 3 | --Invert Sugar | Yes | Invert sugar, usually used as syrups, are sucrose-based syrups treated with the glycoside hydrolase enzyme invertase, and/or an acid, which splits each sucrose molecule into one glucose and one fructose molecule. It is sweeter than an equivalent sucrose solution by weight and is also more hygroscopic, so it can be used to make a product that stays moist longer compared to sucrose. This property is particularly valued by bakers and is found mainly in baked goods and confectionary as well as tobacco products. |
| 3 | --Inositol | No | Naturally occurring in various cereals, nuts, beans and fruit, inositol is a kind of sugar alcohol that is also classified as a B vitamin (often referred to as vitamin B8). |
| 3 | --Sucrose | Yes | Sucrose or table sugar is a disaccharide extracted from sugar cane or sugar beet. It is still the most widely used sweetener in the food and drinks industry, with key product categories including soft drinks, confectionary and baked goods. |
| * Included based on an assumed sugar/water ratio of 70/30; in the analyses, only the sugar content was included. Data sources: Euromonitor International (Passport database). | | | |

1. **Comparison with other studies**
   1. **Studies from Germany**

A comparison of the finding of our analysis with those by two reports of Germany’s Federal Institute for Nutrition Research (Max-Rubner-Institut, or MRI) published in 2018 and 2020 is provided in the main manuscript [34, 36].Two further reports on the sugar content of soft drinks in Germany were published by foodwatch, an advocacy group [15, 16]. The reports are based on store audits in Germany’s three biggest food retailers, conducted in 2016 and 2018. The mean sugar content of soft drinks was reported to be 7.5% in 2016, and 7.3% in 2018. The figures were not weighted by sales, and are therefore not directly comparable to ours. Our figures for the mean sales-weighted sugar content are lower for both years (5.27 g/100ml and 5.25 g/100ml, respectively), which may reflect higher relative demand and thus higher relative sales for soft drink varieties with relatively lower sugar contents (which results in lower sales-weighted means, but does not affect the simple mean not weighted by sales).

With regard to sales volumes, the German Soft Drinks Industry Association (WAFG) has published data for 2012-2021 [17] Their definition of soft drinks includes ready-to-drink coffee but excludes nectars, and therefore differs from the one used in our analysis. Sales figures for soft drinks reported by the WAFG are lower than ours (e.g. 118 l/capita/year, equivalent to 324 ml/capita/day in 2021, compared to 389 ml/capita/day in our analysis) [17]. This may reflect the popularity of nectars in Germany.

The most recent population-based, nationally representative dietary surveys in Germany have not differentiated between 100% fruit juice (which is not included in our analysis) and nectars (which are) [18]. Their results are therefore not directly comparable to our findings. For adults, the most recent assessment, done in 2005-2007, found a mean self-reported consumption of 198 g/capita/day for lemonades, and of 225 g/capita/day for fruit juices, juice drinks, and nectars for men, and 90 g/capita day and 197 g/capita/day for women, respectively, [19], which is compatible with our findings.

- 1. **Studies from the UK**

Studies examining sugar content and sugar sales from soft drinks in the UK equally report findings that are compatible with ours. An assessment by Public Health England used home scan data from the Kantar Worldpanel (for which a representative sample of consumers scan the barcodes of products they buy) to assess retail sales, and a variety of non-representative data sources to assess sales in the out of home (or hospitality) sector [20]. For the retail sector, they found that the mean sales-weighted sugar content of beverages subject to the SDIL fell from 3.9 g/100 ml in 2015 to 2.8 g/100 ml in 2018, a decrease of 28.8%. For the hospitality sector, a decrease in the simple (i.e. not sales-weighted) average total sugar content from 6.1 g/100ml in 2017 to 4.4 g/100ml in 2018 is reported, a decrease by 27.2% [20]. Due to the different methodology, these findings are not directly comparable to our, but similar in magnitude (we found a decrease in the mean sales-weighted sugar content of soft drinks from all sectors from 5.3 g/100ml in 2015 to 4.0 g/100ml in 2018, a decrease of 24%).

A further study examined sugar sales from soft drinks in the UK, using a broader definition of soft drinks (including bottled water and 100% fruit juice) and focusing on large international brands which represent 79% of total volume sales of soft drinks in the UK [21]. It found a reduction in per-capita sugar sales from soft drinks by 4.6 g/capita/day, or 30%, between 2015 and 2018. By contrast, we found a decrease by 5.5 g/capita/day, or 26%. With regard to sugar content, the study reports a decrease in sales-weighted mean sugar content of soft drinks from 4.4 g/100 ml in 2015 to 2.9 g/100 ml in 2018, a reduction of 1.5 g/100 ml, or 34% [21]. We found in that time period a similar decrease (-1,3 g/100 ml, -24%), but on a higher overall level, which may reflect the fact that we did not include bottled water in our analysis (except for flavoured and functional water).

A third study also used Kantar Worldpanel home scan data, and compared actual trends in soft drink purchases in the UK one year after the implementation of the SDIL with a counterfactual based on pre-intervention trends [22]. In line with our findings, it found no effects on total soft drinks sales, but a decrease of in total sugar sales from soft drinks per household per week of 29.5 g, or 9.8%, relative to the counterfactual pre-intervention trend [22]. This figure is lower than the figures reported in our and the previously cited analyses, indicating that part of the decrease observed in sugar sales from soft drinks in that time span in the UK is likely to be a reflection of underlying trends, and not caused exclusively by the SDIL.

1. **Generalizability**

Generalizability of our findings to other settings may be limited. Soft drink consumption varies considerably by country, and the effectiveness of voluntary reformulation strategies may depend on a number of factors, including the perceived likelihood of fiscal or regulatory measures when substantial reformulation is not achieved by proactive industry action alone, among others [23]. Likewise, effects of soft drink taxes may vary by tax design, tax rate, and local circumstances such as public acceptance of replacement of sugar by high-intensity sweeteners, among other factors [6].

1. **Data sources used by Euromonitor**
   1. **Germany**

Research sources used by Euromonitor in constructing the datasets used in our analysis. This information was provided by Euromonitor International.

**Soft Drinks Sources 2022: Germany**

Research Sources | 11 Jul 2022

**Official Sources**

Bundesanzeiger

Bundesforschungsanstalt für Ernährung & Lebensmittel

Bundesministerium für Ernährung & Landwirtschaft

Bundesverband der Erfrischungsgetraenkeindustrie

Deutsche Gesellschaft fuer Ernaehrung

Ördergemeinschaft Ökologischer Landbau Berlin-Brandenburg eV

Statistisches Bundesamt

Statistisches Bundesamt - Destatis

**Trade Associations**

Backmittelinstitut

Bioland ökologische Landbau

Biologische Bundesanstalt für Land- & Forstwirtschaft

Bio-Markt Kompakt

Bund ökologische Lebensmittelwirschaft

Bundesverband der Deutschen Süsswaren Industrie

Bundesverband der Hersteller von Löslichem Kaffee e V (BLK)

Bundesverband des Deutschen Getränkefachgrosshandels eV

Bundesverband des Deutschen Lebensmittelhandels eV (BVL)

Bundesverband Naturkost Naturwaren Herstellung & Handel eV

CMA

Das Kaffeeblatt

DEHOGA

Deutsche Bauern Verband

Deutsche Kaffee Verband

Deutsche Teeverband

Deutsche Zöliakie Gesellschaft

Deutscher Fruchthandelsverband eV

Deutscher Teeverband eV

Deutsches Suppen Institute

Diabetes News Media AG

Diabetes Partner

Die Deutsche Gesellschaft für Ernährung

Die Verbraucher Initiative eV

European Fruit Juice Association

European Fruit Juice Association (AIJN)

German Tea Association

German Vending Association (BDV)

Getränke-Ring

Gewerkschaft Nahrung-Genuss-Gaststätten

Infodienst Landwirtschaft

Informationszentrale Deutsches Mineralwasser

International Bottled Water Association

International Federation of Fruit Juice Producers

Kaffeeverband

Milchindustrie Verband eV

Planet Wissen

PLMA

Sweets Global Network

Tea & Herbal Infusions Europe

UNESDA & CISDA

University Kassel

Vending Trade Association

Verband der deutschen Fruchtsaft-Industrie eV (VdF)

Verband der Ernährung & Diätetik eV

Verband Deutscher Großbäckereien eV

Verband deutscher Mineralbrunnen

Verband deutscher Mineralbrunnen (VDM)

Verband Deutscher Mineralbrunnen eV

WAFG

Wirtschaftsvereinigung Alkoholfrei Getränke eV

Wirtschaftsvereinigung Alkoholfreie Getränke (WAFG)

Wirtschaftsvereinigung Kräuter & Früchtetee eV

Wirtschaftsvereinung Alkoholfreie Getränke

ZMP

**Trade Press**

About Drinks

Beverage Net

Beverage World

Bio Markt Info

Bio Press

Bundesverband Systemgastronomie

CASH Das Handelsmagazin

CHD Expert (Deutschland) GmbH

Cocoabarometer

Deutsche Getränke Wirtschaft

Deutscher Fachverlag

Deutscher Landwirtschaftsverlag GmbH

Die Welt

Espresso International

Fluessiges Obst

Food & Drink Europe

Food News

Gastgewerbe Magazin

Gastronomie Magazine

Gesundheit.de

Getränke New

Getränke News

Getränkefachgroßhandel

Getränkemarkt

Grips & Co

Handelsblatt

HGV Praxis

Horizont.net

Infozentrum Schokolade

Inside

Inside Jun2021

KarmaKonsum

Konsumo

Lebensmittel Praxis

Lebensmittelzeitung

LZ Mineralbrunnen Ranking Jun2021

Markant Handelsmagazin

Markenartikel

Meininger Online

Naturkost.de

Netdoctor.de

Oko Markt

Osnabruecker Zeitung

Sachon Fachzeitschriftenarchive

Soft Drinks International

Stiftung Warentest

STIR

Szene drinks

Tea & Coffee Trade Journal

Welt.de

Zöliakie Treff

**Company Sources**

Adelholzener Alpenquellen GmbH

ADM Wild Europe GmbH & Co KG

Al Natura

Albi GmbH & Co KG

Aldi-Süd

Allos

Alnatura Produktions & Handels GmbH

Alois Dallmayr KG

Andechser Molkerei Scheitz GmbH

Apollinaris & Schweppes GmbH

Appel Feinkost GmbH & Co KG

Arla Foods GmbH

asianbrand.de

Asinto Getranke GmbH

Azuco

Bad Heilbrunner Reform-Diät-Arznei GmbH & Co

Baerenmarke

Bahlsen GmbH Co & KG

Bauer KG, J

Beckers Bester GmBH

Bell Flavours & Fragrances GmbH

Berentzen Gruppe Aktiengesellschaft

Beyers Koffie

Bionade GmbH

Bio-Wertkost

Bittenfelder

Bonduelle GmbH

Brau & Brunnen AG

Breisgaumilch GmbH

Brita

Bünting Teehandelshaus GmbH & Co, J

Cafeahaus AG

Campina GmbH

Castle Tea Co GmbH

CFP Brands Süßwarenhandels GmbH & Co KG

Coca-Cola GmbH

Coffeevent

Dallmayr Group

Danone Waters Deutschland GmbH

Danone, Groupe

Darboven GmbH & Co JJ

DE Master Blenders

Demeter Marktforum eV

Destilla GmbH

Deutsche Extrakt Kaffee GmbH

Deutsche Sinalco GmbH Markengetränke & Co KG

Deutsche SiSi-Werke GmbH & Co KG

Deutscher Kaffee Verband eV

Develey

Die Farüchoc Schokoladenfabrik

Die Krüger GmbH & Co KG

Döhler GmbH

Dr August Oetker Nahrungsmittel KG

Dr Goerg

Dr Schär GmbH

Drinks & More GmbH & Co KG

Drinkstar GmbH

Eckes Granini

Eckes-Granini GmbH

Elka Frische GmbH

Emmi Deutschland GmbH

Euryza GmbH

Ferrero GmbH

Franken Brunnen GmbH & Co KG

Frispa GmbH

Frosta AG

Fruit2day

FSP GmbH

Füllhorn

Fürsten-Reform Dr Med Hans Plümer Nachf GmbH & Co

GEDAT Getränkedaten GmbH

Genuport (Isostar)

Genuport Trade GmbH

GEPA - The Fair Trade Co

Gerolsteiner

Gondwana Trade Grundstück & Verwaltung GmbH & CO KG

Gourmesso

Grünes Land

H&S Teegeselschaft

Hamburg Tea

Hans Döhle GmbH

Hassia Gruppe

Hassia Mineralquellen GmbH & Co KG

Herza Schokolade GmbH & Co KG

Hipp GmbH & Co Vertrieb KG

Hochwald Foods GmbH

IGlanbia Nutritionals

Illy Caffé SpA

Immo Eifler

Importhaus Wilms

Innoprax GmbH

Intersnack Knabber-Gebäck GmbH & Co KG

J Bünting Teehandelshaus GmbH & Co

Jacobs Douwe Egberts DE GmbH

Jacobs Douwe Egberts LLC

JJ Darboven GmbH & Co

Kampffmeyer AG

Kauf-idee

K-Fee AG

K-fee System GmbH

KHR Food Import GmbH

King Car Germany GmbH

Klindworth Fruchtsäfte GmbH

Klosterfrau GmbH

Kraft Foods Deutschland GmbH

Krings Fruchsaft

Krüger GmbH & Co KG

Kulau GmbH

Laurens Spethmann Holding AG & Co

Lavazza Deutschland GmbH

Lavazza SpA, Luigi

Lichtenauer Mineralquellen GmbH

Lieken AG

Lindt & Sprüngli AG

Lorenz Bahlsen Snack-World GmbH & Co KG Germany, The

Manuli

Marc & Monkey's GmbH

Markengetränke Schwollen GmbH

Martin Bauer Group

Masterfoods GmbH

MatchaMagic International

Maxingvest AG

May Milch GmbH & Co KG

Mayka Naturbackwaren GmbH

MBG International Premium Brands GmbH

Melitta

Melitta Europa GmbH & Co KG

Melitta Gruppe

Merziger Fruchtgetränke GmbH

Mineralbrunnen-Üeberkingen-Teinech GmbH & Co KG

Molkerei Alois Müller GmbH & Co KG

Mondelez International Inc

Monster Energy Co

Mount Everest Tea GmbH

Natumi AG

Naturata AG

Naturkind

Nespresso Deutschland GmbH

Nestlé Deutschland AG

Nestlé Waters Deutschland AG

Niehoffs Vaihinger Fruchtsaft GmbH

Oasis Teehandel

Oberland Milchverwertung Ravensburg GmbH

Oekoinform

Ökoland GmbH Nord

OMIRA Oberland Milchverwertung GmbH

Optima Packaging GmbH

OPTIMA packaging group GmbH

Ostfriesische Tee Gesellschaft GmbH & Co KG

PepsiCo Deutschland GmbH

Pfanner GmbH

Power Horse Energy Drink GmbH

Pressebüro Melitta c/o Emanate GmbH

Punica

Quaker Beverages GmbH

Querfood.com

Rapunzel Naturkost AG

Rauch Deutschland GmbH & Co KG

Rauch Fruchtsäfte GmbH & Co KG

Red Bull GmbH

Rheinfelsquelle H Hövelmann GmbH & Co KG

Rhönsprudel

Riha.de

Robert Koch Institut Journal of Health Monitoring

San Pellegrino

Sara Lee Deutschland GmbH

Schneekoppe GmbH & Co KG

Schuster's Shrub GmbH

Schwartauer Werke GmbH & Co KGaA

Schweppes Deutschland GmbH

Sektkellerei Bernard-Massard

Shark Energy Drink

Sinass-Tee

Sinziger Mineralbrunnen GmbH

SLE Innovation GmbH

SmartCon

SodaStream GmbH

Spezi

Splendid Drinks AG

Sportfit

Statistisches Bundesamt DeStatis

Stick & Lembke GmbH

Süßwarenhandels GmbH & Co KG

Tchbio GmbH

Tchibo Frisch-Röst-Kaffee GmbH

Tchibo GmbH

TeeGschwendner GmbH

Teekanne GmbH & Co KG

Thiele

Trendworx GmbH

Tri Top GmbH

TSI GmbH&Co

Tucano Fruchtsaft

Twining & Co Ltd

Unilever Deutschland GmbH

Union Deutsche Lebensmittelwerke GmbH

Valensina GmbH

Velibre

Verband der deutschen Fruchtsaftindustrie

Vilsa-Brunnen Otto Rodekohr GmbH & Co KG

Vitamizzer GmbH

VIVIL A Müller GmbH & Co KG

Wagner Tiefkühlprodukte GmbH

Weight Watchers Germany

Wertform GmbH

Wrigley GmbH

Yogi Tea GmbH

Zeekei GmbH

Zentrale Markt & Preisberichtstelle GmbH (ZMP)

Zimbo GmbH & Co KG

**Other Sources**

Cholestrin Senken

Convenience Shop

Deutsche Wellness Verband

Diätverband eV

Dr-q.de

Eis Eis Info Service

Factiva

Focus Marktanalysen

Grosseinkaufsring des Süsswaren & Getränkehandels (GES)

Hanzsch Kommunikation

Isostar.de

Just-drinks.com

Kaffee-kapseln.biz

kapsel-kaffee.net

kapselmaschinen.net

Müngersdorf Laden

Natreen

Presseportal

Seismoblog - Natur & Umwelt

Seven One Media

Silver-arrow.de

Soda Club GmbH

Süddeutsche Zeitung

TransFair

- 1. **UK**

Research sources used by Euromonitor in constructing the datasets used in our analysis. This information was provided by Euromonitor International.

**Soft Drinks Sources 2022: United Kingdom**

Research Sources | 11 Jul 2022

**Official Sources**

Centre for the Promotion of Importation (CBI)

Gov UK

Results Publication

**Trade Associations**

Automatic Vending Association

Bakers Federation

British Bottled Water Association

British Coffee Association

British Soft Drinks Association

BSDA

Copella Fruit Juices

Food & Drink Federation

Food Ingredients Europe

Food Standards Agency

Home Grown Cereals Authority

International Bottled Water Association

International Coffee Organisation

International Federation of Fruit Juice Producers

International Tea Committee, The

Milk Development Council

Natural Mineral Water Association

Organic Milk Suppliers Cooperative

Organic Trade Board

Soil Association

Speciality Coffee Association of Europe (SCAE)

Specialty Coffee & Tea Association

Tea Council

UK Herbal Infusions Association

UNESDA & CISDA

**Trade Press**

Beverage Daily

Beverage Net

Beverage World

Bloomberg

Brand Republic

Checkout

Chemist & Druggist

Convenience Store

Daily business

Dairy Reporter

Firefly Juice/Tonics

Food & Drink Innovation Network

Food & Drink International

Food Magazine

FoodBev.com

Foodnavigator.com

Grazia

International Herald & Tribune

Just-food.com

Licensing World

Mad.co.uk

Marketing

Morning Advertiser

Packaging Europe

Packaging News

Real Coffee Magazine

Soft Drinks International

Talking Retail

The Drinks Business

The Financial News

The Grocer

The Guardian

The Independent

The Publican

The Telegraph

The Times

**Company Sources**

ADM Milling Ltd

AG Barr Plc

Aimia Foods

All Market Europe Ltd

Allied Bakeries

Andechser Lassi

Arctic Iced Coffee

Asda Plc

Baker Perkins Ltd

Barr (AG) Plc

Bella Berry

Belvoir

Belvoir Fruit Farms

Betty & Taylor's of Harrogate

Beverage Brands (UK) Ltd

BigShit NI Ltd

Bio-Synergy UK

Boost Drinks

Bottlegreen Drinks Co

Bravura Foods Ltd

British Bakels Ltd

British Bakeries Ltd

Britvic

Britvic EMEA

Britvic Plc

Café Pod

Cafédirect Plc

CaféPod Ltd

Caffe Nero

Calypso Soft Drinks Ltd

Campden BRI

Cereform Ltd

Clipper Teas Ltd

Coca-Cola Co, The

Coca-Cola Enterprises Ltd

Coldpress

Cool Cold Brew

Co-op

Costa Coffee

Cott Beverages Ltd

Crussh

Cushiedoos

Danone Waters UK

Danone, Groupe

DEFRA

Del Monte Europe Ltd

Del Monte Foods UK Ltd

Délifrance

Dorset Cereals

Douwe Egberts

Dr Pepper Snapple Group

DR Wakefield & Co Ltd

Duchy Originals

Edme Ltd

Emerging Traders Ltd

Emmi UK Ltd

Equinox Kombucha

Feel Good Drinks Co

Feelfine

Fentimans Ltd

Fever Tree

Fine Foods International

Fine Lady Bakeries

Fiovana Soft Drinks

Fitch Brew Co

Food Brands Group Ltd

Forever Young Ltd

Forum for the Future

Franklin & Sons Ltd

Frucor Beverages Ltd

Frutarom

Fuerst Day Lawson

GB Ingredients

Gerber Soft Drinks Ltd

Glanbia Plc

GlaxoSmithKline Plc

Gococo

Good Culture Kombucha

GoodnessDirect

Grant Solutions BV

Grove Fresh

Gusto Drinks

Gusto Organics Ltd

Harrogate Spring Water Ltd

Hartridges

Heartland Food Products Group LLC

Heinz Ltd

Highland Spring Ltd

Horlicks

Illy

Illy Caffé SpA

IMA Packaging

Innocent Drinks Ltd

Inside Out Beauty - SIP Water

Irwins Bakery

Jacobs Douwe Egberts LLC

James White Drinks

JARR Kombucha

JDE Coffee - Tassimo

JDE Peet's BV

Jing Tea

Johnsons Juice

Jordan Cereals Ltd

Juice Doctor

Kanegrade Ltd

Kenco

Kenwood Ltd

Koninklijke Philips Electronics NV

Kraft Foods Inc

Lavazza Coffee (UK) Ltd

Lavazza SpA, Luigi

Lesaffre Group

Lidl

Lishi Tea

Little Miracles

Loon Fung Ltd

Lucozade Suntory Ribena Ltd

Lucozade UK

Mangajo

Marks & Spencer Plc

Marley Coffee

Marlow Foods Ltd

Moju Drinks

Mondelez International Inc

Monster

Morrison Supermarkets plc

Musgrave Budgens Londis

Nasa shop owner

Natural Beverages

Nespresso

Nestlé UK Ltd

Nestlé Waters

Nestlé Waters Group

Nichols Foods Plc

Nothing But Tea

Novartis AG

Nutricia Co Ltd

Ocean Spray Cranberries Inc

OKF Co Ltd

Orangina Group

Orchard House

Pago Fruit Juices

PepsiCo International Inc

Percol Coffee

Plenish

POW Energy Water

Powerade

Powershot

Premier Foods Plc

Princes

Princes Ltd

Procter & Gamble Co, The

Puratos Ltd

Pussy Drinks

Qcumber

RDA Organic

Real Coffee Bean Co, The

Real Kombucha

Red Bull

Red Bull Co Ltd

Red Bush Tea Co

Redwood Wholefood Co Ltd, The

RJA Foods - Pomegreat

Roberts Bakery

Rowse Honey Ltd

Rubicon Drinks Ltd

Sainsbury plc, J

Sara Lee Corp

Scheckters Organic

Schweppes UK

SHS Drinks

Sidel Packaging

Silver Spring Mineral Water Co

Solution Sciences Ltd

Spadel UK Ltd

Starbucks Corp

Starbucks UK

Stir Tea & Coffee

Strathmore Mineral Water

Stute Foods

Sunjuice Ltd

Sunraysia Natural Beverage Co, The

Sunsweet

Tata Global Beverages Ltd

Taylors of Harrogate

Tea Experience, The

Teapigs

Tenzing

Tesco Plc

Tetley UK

Thai Coco

Tick Tock Tea

T-Plus Drinks

Tropicana UK Ltd

Trustin Unimerchants

Twining & Co Ltd

Ty Nant

Typhoo Ltd

UCC Coffee

UCC Coffee UK Ltd

Unilever Group

Unilever UK plc

Unococo

Valio Oy

Vandemoortele Group

Vegesentials Ltd

Vimto Drinks

Virtue Drinks

Vita Coco

Waitrose

Warburtons Ltd

Weanie Beans

Well Well Well

Wellness Quest

Whitbread

William Jackson & Son Ltd

Yoplait UK Ltd

Zeelandia Ltd

**Other Sources**

Allegra Strategies

Allegra World Coffee Portal

Ceoliac Society

Factiva

Financial Statement

Financial Times

Foodmanufacture.co.uk

GranoVita Ltd

Just-drinks.com

MySupermarket

Ocado.com

Pennine Tea & Coffee Ltd

Strategy Business

1. **Differences between protocol and manuscript**

This study is based on a protocol developed and prospectively registered and published through the Open Science Framework (registration DOI 10.17605/OSF.IO/3WJ49) before data was analysed [24]. In the following, we descibe differences between the protocol and the manuscript:

- We decided to include a comparison between the trend in the mean sales-weighted sugar content of soft drinks sold in Germany with reduction targets set by Germany’s national sugar reduction strategy, to allow for a direct comparison between actual trends and government targets. Besides, we also included a comparisons of trends before and after the national sugar reduction strategy was announced.
- We decided to frame the article as an evaluation of this strategy, in order to highlight its policy implications.
- We decided to include concentrates in our analysis of sugar sales from soft drinks per capita, but not in the analysis of mean sales-weighted sugar content and soft drinks sales volume per capita, as dilution ratios may vary.
- We decided to apply a multiplier of 0.7 to ingredient volume figures for glucose-fructose syrup, glucose/corn syrup and high-fructose corn syrup, to account for the water content of these.
- We decided to replace the total amount of sugar sold through soft drinks in tonnes/year by sugar sales from soft drinks per capita reported in g/capita/day, as we found these figures easier to interpret for public health purposes.

# **STROBE-nut Checklist**

The STROBE-nut reporting guideline checklist is available online at: <https://www.strobe-nut.ugent.be/content/recommendations>

Page numbers with a preceding s (s1, s2, etc.) refer to the supplementary annexe.

| **eTable 4: STROBE-nut Checklist** | | | | |
| --- | --- | --- | --- | --- |
| **Nr** | **Item** | **STROBE recommendations** | **STROBE-nut** | **Page Nr** |
| 1 | **Title and**  **abstract** | (a) Indicate the study’s design with a commonly used term in the title or the abstract.  (b) Provide in the abstract an informative and balanced summary of what was done and what was found. | **nut-1**State the dietary/nutritional assessment method(s) used in the title, abstract, or keywords. | 1, 2 |
|  | **Introduction** |  |  |  |
| 2 | Background    rationale | Explain the scientific background and rationale for the investigation being reported. |  | 3 |
| 3 | Objectives | State specific objectives, including any pre-specified hypotheses. |  | 3,4 |
|  | **Methods** |  |  |  |
| 4 | Study design | Present key elements of study design early in the paper. |  | 4 |
| 5 | Settings | Describe the setting, locations, and relevant dates, including periods of recruitment, exposure, follow-up, and data collection. | **nut-5** Describe any characteristics of the study settings that might affect the dietary intake or nutritional status of the participants, if applicable. | 4 |
| 6 | Participants | a) Cohort study—Give the eligibility criteria, and the sources and methods of selection of participants. Describe methods of follow-up.  Case-control study—Give the eligibility criteria, and the sources and methods of case ascertainment and control selection. Give the rationale for the choice of cases and controls.  Cross-sectional study—Give the eligibility criteria, and the sources and methods of selection of participants.  (b) Cohort study—For matched studies, give matching criteria and number of exposed and unexposed.  Case-control study—For matched studies, give matching criteria and the number of controls per case. | **nut-6** Report particular dietary, physiological or nutritional characteristics that were considered when selecting the target population. | 4-5 and s2-s5 |
| 7 | Variables | Clearly define all outcomes, exposures, predictors, potential confounders, and effect modifiers. Give diagnostic criteria, if applicable. | **nut-7.1**Clearly define foods, food groups, nutrients, or other food components.  **nut-7.2** When using dietary patterns or indices, describe the methods to obtain them and their nutritional properties. | 4-5 and s5-s11 |
| 8 | Data sources - measurements | For each variable of interest, give sources of data and details of methods of assessment (measurement). Describe comparability of assessment methods if there is more than one group. | **nut-8.1** Describe the dietary assessment method(s), e.g., portion size estimation, number of days and items recorded, how it was developed and administered, and how quality was assured. Report if and how supplement intake was assessed.  **nut-8.2** Describe and justify food composition data used. Explain the procedure to match food composition with consumption data. Describe the use of conversion factors, if applicable.  **nut-8.3** Describe the nutrient requirements, recommendations, or dietary guidelines and the evaluation approach used to compare intake with the dietary reference values, if applicable.  **nut-8.4** When using nutritional biomarkers, additionally use the STROBE Extension for Molecular Epidemiology (STROBE-ME). Report the type of biomarkers used and their usefulness as dietary exposure markers.  **nut-8.5** Describe the assessment of nondietary data (e.g., nutritional status and influencing factors) and timing of the assessment of these variables in relation to dietary assessment.  **nut-8.6** Report on the validity of the dietary or nutritional assessment methods and any internal or external validation used in the study, if applicable. | 4-5 and s4-s5 |
| 9 | Bias | Describe any efforts to address potential sources of bias. | **nut-9** Report how bias in dietary or nutritional assessment was addressed, e.g., misreporting, changes in habits as a result of being measured, or data imputation from other sources | s4-s5 |
| 10 | Study Size | Explain how the study size was arrived at. |  | S2 |
| 11 | Quantitative    variables | Explain how quantitative variables were handled in the analyses. If applicable, describe which groupings were chosen and why. | **nut-11** Explain categorization of dietary/nutritional data (e.g., use of N-tiles and handling of nonconsumers) and the choice of reference category, if applicable. | 4-5 and s4-s5 |
| 12 | Statistical              Methods | (a) Describe all statistical methods, including those used to control for confounding  (b) Describe any methods used to examine subgroups and interactions.  (c) Explain how missing data were addressed.  (d) Cohort study—If applicable, explain how loss to follow-up was addressed.  Case-control study—If applicable, explain how matching of cases and controls was addressed.  Cross-sectional study—If applicable, describe analytical methods taking account of sampling strategy.  (e) Describe any sensitivity analyses. | **nut-12.1** Describe any statistical method used to combine dietary or nutritional data, if applicable.  **nut-12.2** Describe and justify the method for energy adjustments, intake modeling, and use of weighting factors, if applicable.  **nut-12.3** Report any adjustments for measurement error, i.e,. from a validity or calibration study. | 4-5 and s4-s5 |
|  | **Results** |  |  |  |
| 13 | Participants | (a) Report the numbers of individuals at each stage of the study—e.g., numbers potentially eligible, examined for eligibility, confirmed eligible, included in the study, completing follow-up, and analyzed.  (b) Give reasons for non-participation at each stage.  (c) Consider use of a flow diagram. | **nut-13** Report the number of individuals excluded based on missing, incomplete or implausible dietary/nutritional data. | n.a. |
| 14 | Descriptive data | (a) Give characteristics of study participants (e.g., demographic, clinical, social) and information on exposures and potential confounders  (b) Indicate the number of participants with missing data for each variable of interest  (c) Cohort study—Summarize follow-up time (e.g., average and total amount) | **nut-14** Give the distribution of participant characteristics across the exposure variables if applicable. Specify if food consumption of total population or consumers only were used to obtain results. | n.a. |
| 15 | Outcome data | Cohort study—Report numbers of outcome events or summary measures over time.  Case-control study—Report numbers in each exposure category, or summary measures of exposure.  Cross-sectional study—Report numbers of outcome events or summary measures. |  | 5-6, Table 1 and Fig. 1-3 |
| 16 | Main results | (a) Give unadjusted estimates and, if applicable, confounder-adjusted estimates and their precision (e.g., 95% confidence interval).  Make clear which confounders were adjusted for and why they were included.  (b) Report category boundaries when continuous variables were categorized.  (c) If relevant, consider translating estimates of relative risk into absolute risk for a meaningful time period. | **nut-16** Specify if nutrient intakes are reported with or without inclusion of dietary supplement intake, if applicable. | n.a. |
| 17 | Other analyses | Report other analyses done—e.g., analyses of subgroups and interactions and sensitivity analyses. | **nut-17**Report any sensitivity analysis (e.g., exclusion of misreporters or outliers) and data imputation, if applicable. | n.a. |
|  | **Discussion** |  |  |  |
| 18 | Key results | Summarize key results with reference to study objectives. |  | 6-7 |
| 19 | Limitation | Discuss limitations of the study, taking into account sources of potential bias or imprecision. Discuss both direction and magnitude of any potential bias. | **nut-19** Describe the main limitations of the data sources and assessment methods used and implications for the interpretation of the findings. | 7 |
| 20 | Interpretation | Give a cautious overall interpretation of results considering objectives, limitations, multiplicity of analyses, results from similar studies, and other relevant evidence. | **nut-20** Report the nutritional relevance of the findings, given the complexity of diet or nutrition as an exposure. | 6-8, s12-s14 |
| 21 | Generalizability | Discuss the generalizability (external validity) of the study results. |  | s13 |
|  | **Other information** |  |  |  |
| 22 | Funding | Give the source of funding and the role of the funders for the present study and, if applicable, for the original study on which the present article is based. |  | 9 |
|  | *Ethics* |  | **nut-22.1**Describe the procedure for consent and study approval from ethics committee(s). | 8 |
|  | *Supplementary material* |  | **nut-22.2** Provide data collection tools and data as online material or explain how they can be accessed. | 10 |

# **References**

1. WHO. Fiscal policies for diet and the prevention of noncommunicable diseases. 2016; (November 21, 2019). Available from <https://apps.who.int/iris/bitstream/handle/10665/250131/9789241511247-eng.pdf;jsessionid=945FF538F803E6209AD4F426ED94E6E3?sequence=1>.

2. Obesity Evidence Hub. Countries that have taxes on sugar-sweetened beverages (SSBs). 2022; (July 30, 2022). Available from <https://www.obesityevidencehub.org.au/collections/prevention/countries-that-have-implemented-taxes-on-sugar-sweetened-beverages-ssbs>.

3. Thow AM, Rippin HL, Mulcahy G, Duffey K, Wickramasinghe K. Sugar-sweetened beverage taxes in Europe: learning for the future. European Journal of Public Health. 2022;32(2):273-80.

4. UNC. Sugary drink taxes around the world (Updated May 2022). 2022; (July 30, 2022). Available from <https://www.globalfoodresearchprogram.org/resources/maps/>.

5. WCRFI. NOURISHING and MOVING policy databases. 2022; (July 30, 2022). Available from <https://policydatabase.wcrf.org/>.

6. WHO Regional Office for Europe. Sugar-sweetened beverage taxes in the WHO European Region: success through lessons learned and challenges faced. 2022; (July 31, 2022). Available from <https://apps.who.int/iris/handle/10665/351781>.

7. HM Treasury. Budget 2016. 2016 2018-07-14.

8. Euromonitor International. Passport. 2022; (July 31, 2022). Available from <https://www.euromonitor.com/our-expertise/passport>.

9. WHO. World Health Organization. Sugars intake for adults and children. 2015; (December 8, 2019). Available from <http://apps.who.int/iris/bitstream/10665/149782/1/9789241549028_eng.pdf?ua=1>.

10. BMJ. Verordnung über einige zur menschlichen Ernährung bestimmte Zuckerarten (Zuckerartenverordnung), Anlage 1 (zu den §§ 1 bis 4) - Bezeichnungen und Begriffsbestimmungen. 2003 September 2, 2022. Available from <http://www.gesetze-im-internet.de/zuckartv_2003/anlage_1.html>.

11. Suedzucker. Glucose-Fructose-Sirup. 2022 September 3, 2022. Available from <https://www.suedzucker.com/de/product/glukose-fruktose-sirup/>.

12. World Bank. Population, total. 2020; (July 31, 2022). Available from <https://data.worldbank.org/indicator/SP.POP.TOTL>.

13. Max-Rubner-Institut. Zuckergehalte von zuckergesüßten Erfrischungsgetränken: Differenzierung von Produktuntergruppen und Berechnung von Quartilen. 2018 September 3, 2018. Available from <https://www.mri.bund.de/fileadmin/MRI/Themen/Reformulierung/180911_Bericht_Zuckergehalt_Erfrischungsgetraenke-Titel.pdf>.

14. Max-Rubner-Institut. Ergebnisbericht Produktmonitoring 2019 (Version 2.0, Juni 2020). 2020; (October 19, 2022). Available from <https://www.mri.bund.de/fileadmin/MRI/Institute/EV/Produktmonitoring-2019_Ergebnisbericht-Version-2.0.pdf>.

15. foodwatch. foodwatch Marktstudie 2016: So zuckrig sind "Erfrischungsgetränke" in Deutschland. 2016 2017-05-21. Available from <http://www.foodwatch.org/uploads/media/Marktstudie_final_WEB_04.pdf>.

16. foodwatch. So zuckrig sind "Erfrischungsgetränke" in Deutschland - immer noch. 2018 September 3, 2022. Available from <https://www.foodwatch.org/fileadmin/foodwatch.de/news/2018-09-21_foodwatch-Marktstudie-Zuckergetraenke_01.pdf>.

17. WAFG. Entwicklung des Pro-Kopf-Verbrauchs von Alkoholfreien Getränken nach Getränkearten 2012 - 2021. 2022; (October 19, 2022). Available from <https://www.wafg.de/fileadmin/dokumente/pro-kopf-verbrauch.pdf>.

18. von Philipsborn P, Hauck C, Gatzemeier J, Landsberg B, Holzapfel C. Süßgetränke und Körpergewicht: Zusammenhänge und Interventionsmöglichkeiten. Adipositas. 2017;11(3):140-5.

19. Max-Rubner-Institut. Nationale Verzehrstudie II - Lebensmittelverzehr und Nährstoffzufuhr auf Basis von 24-h-Recalls. 2013.

20. Public Health England. Sugar reduction: Report on progress between 2015 and 2018. 2019 September 3, 2022. Available from <https://assets.publishing.service.gov.uk/government/uploads/system/uploads/attachment_data/file/839756/Sugar_reduction_yr2_progress_report.pdf>.

21. Bandy LK, Scarborough P, Harrington RA, Rayner M, Jebb SA. Reductions in sugar sales from soft drinks in the UK from 2015 to 2018. BMC Medicine. 2020 2020/01/13;18(1):20.

22. Pell D, Mytton O, Penney TL, Briggs A, Cummins S, Penn-Jones C, et al. Changes in soft drinks purchased by British households associated with the UK soft drinks industry levy: controlled interrupted time series analysis. BMJ. 2021;372:n254.

23. Sharma LL, Teret SP, Brownell KD. The food industry and self-regulation: standards to promote success and to avoid public health failures. American journal of public health. 2010 Feb;100(2):240-6.

24. von Philipsborn P. Taxation, consumption and sugar content of sugar-sweetened beverages in Germany and the United Kingdom, 2011-2021: a comparative analysis. OSF Registries. 2022 August 20, 2022. Available from <https://osf.io/3wj49>.
